# ISOlation Procedure vs. conventional procedure during Distal Pancreatectomy (ISOP-DP trial): study protocol for a randomized controlled trial

**DOI:** 10.1186/s13063-021-05523-y

**Published:** 2021-09-16

**Authors:** Ken-ichi Okada, Manabu Kawai, Seiko Hirono, Masayuki Sho, Masaji Tani, Ippei Matsumoto, Suguru Yamada, Ryosuke Amano, Hirochika Toyama, Yo-ichi Yamashita, Takeshi Gocho, Kazuto Shibuya, Minako Nagai, Hiromitsu Maehira, Keiko Kamei, Go Ohira, Yoshihiro Shirai, Hideki Takami, Nana Kimura, Takumi Fukumoto, Hideo Baba, Yasuhiro Kodera, Akimasa Nakao, Toshio Shimokawa, Masahiro Katsuda, Hiroki Yamaue

**Affiliations:** 1 Second Department of Surgery, School of Medicine, Wakayama Medical University, 811-1 Kimiidera, Wakayama, 641-8510 Japan; 2 Department of Surgery, Nara Medical University, Kashihara, Japan; 3 Department of Surgery, Shiga University of Medical Science, Ōtsu, Japan; 4 Department of Surgery, Kindai University Hospital, Osaka, Japan; 5 Department of Surgery, Nagoya Central Hospital, Nagoya, Japan; 6 Department of Hepato-Biliary-Pancreatic Surgery, Graduate School of Medicine, Osaka City University, Osaka, Japan; 7 Department of Surgery, Division of Hepato-Biliary-Pancreatic Surgery, Graduate School of Medicine, Kobe University, Kobe, Japan; 8 Department of Gastroenterological Surgery, Graduate School of Medical Sciences, Kumamoto University, Kumamoto, Japan; 9 Department of Surgery, Jikei University School of Medicine, Tokyo, Japan; 10 Department of Surgery and Science, Faculty of Medicine, Academic Assembly, University of Toyama, Toyama, Japan; 11 Department of Gastroenterological Surgery, Graduate School of Medicine, Nagoya University, Nagoya, Japan; 12 Clinical Study Support Center, Wakayama Medical University, Wakayama, Japan

**Keywords:** Distal pancreatectomy, Distal pancreatosplenectomy, Isolation, Radical antegrade modular pancreatosplenectomy

## Abstract

**Background:**

Radical antegrade modular pancreatosplenectomy (RAMPS) is an isolation procedure in pancreatosplenectomy for pancreatic body/tail cancer. Connective tissues around the bifurcation of the celiac axis are dissected, followed by median-to-left retroperitoneal dissection. This procedure has the potential to isolate blood and lymphatic flow to the area of the pancreatic body/tail and the spleen to be excised. This is achieved by division of the inflow artery, transection of the pancreas, and then division of the outflow vein in the early phases of surgery. In cases of pancreatic ductal adenocarcinoma (PDAC), the procedure has been shown to decrease intraoperative blood loss and increase R0 resection rate by complete clearance of the lymph nodes. This trial investigates whether the isolation procedure can prolong the survival of patients with pancreatic ductal adenocarcinoma who undergo distal pancreatosplenectomy (DPS) compared with those that undergo the conventional approach.

**Methods/design:**

Patients with PDAC scheduled to undergo DPS are randomized before surgery to undergo either a conventional procedure (arm A) or to undergo the isolation procedure (arm B). In arm A, the pancreatic body, tail, and spleen are mobilized, followed by removal of the regional lymph nodes. The splenic vein is transected at the end of the procedure. The timing of division of the splenic artery (SA) is not restricted. In arm B, regional lymph nodes are dissected, then we transect the root of the SA, the pancreas, then the splenic vein. At the end of the procedure, the pancreatic body/tail and spleen are mobilized and removed. In total, 100 patients from multiple Japanese high-volume centers will be randomized. The primary endpoint is 2-year recurrence-free survival by intention-to-treat analysis. Secondary endpoints include intraoperative blood loss, R0 resection rate, and overall survival.

**Discussion:**

If this trial shows that the isolation procedures can improve survival with a similar R0 rate and with a similar number of lymph node dissections to the conventional procedure, the isolation procedure is expected to become a standard procedure during DPS for PDAC. Conversely, if there were no significant differences in endpoints between the groups, it would demonstrate justification of either procedure from surgical and oncological points of view.

**Trial registration:**

UMIN Clinical Trials Registry UMIN000041381. Registered on 10 August 2020. ClinicalTrials.gov NCT04600063. Registered on 22 October 2020.

**Supplementary Information:**

The online version contains supplementary material available at 10.1186/s13063-021-05523-y.

## Background

Several investigators have advocated the possibility that grasping tumors during pancreatectomy may increase the risk of squeezing and thus shedding cancer cells into the portal vein, retroperitoneum, and/or peritoneal cavity [1, 2]. To overcome these problems, a number of surgical techniques have been proposed, aiming for non-touch pancreatectomy [3].

Many gastroenterological surgeons have tried to demonstrate oncological benefits of such non-touch techniques, similar in concept to the isolation technique for resection of several gastrointestinal cancers. The impact of the isolation technique has not yet been demonstrated by scientifically acceptable methods [3, 4]. Considering the portal venous system as a drainage vein of the pancreatic body/tail and spleen, we suggest the anatomical features may be suitable for verification of the oncological benefits of the isolation technique.

Radical antegrade modular pancreatosplenectomy (RAMPS) was reported by Strasberg et al. in 2003 as a new antegrade procedure which provides improved visibility and allows removal of N1 nodes. It permits adjustment of the depth of the posterior extent of resection coupled with early rather than late control of the vasculature. Descriptions of the RAMPS procedure indicate the potential of isolation of blood and lymphatic flow to the area of the pancreatic body/tail and spleen to be excised [5, 6]. This is achieved by dividing the inflow artery, transecting the pancreas, and dividing the outflow vein in the early phases of surgery.

The RAMPS method has since been reported to have oncological usefulness as a favorable method for each lymph node dissection and R0 resection in multiple institutions [7–12], but all of these studies have been retrospective historically controlled studies. RAMPS performed in recent years, with the development of surgical techniques and devices, has been compared with the conventionally used procedures. No randomized controlled studies have yet compared the clinical usefulness of the isolation procedure with the conventional procedures as a control group in distal pancreatosplenectomy (DPS) for patients with pancreatic ductal adenocarcinoma (PDAC).

To confirm the results of the cohort study, we will conduct a multicenter, randomized, placebo-controlled phase II trial of the isolation procedure (ISOP-DP trial). We will evaluate whether it affects the recurrence-free survival (RFS) rate compared with conventional procedures after DPS. This trial is registered at the UMIN Clinical Trials Registry (UMIN000041381) and at ClinicalTrials.gov (NCT04600063).

## Methods/design

### Study design and overview

**Fig. 1:**
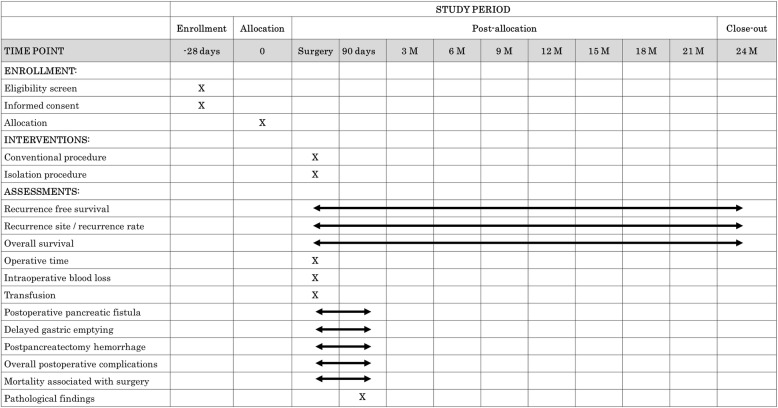
Study calendar

The ISOP-DP trial is a Japanese multicenter, randomized, controlled trial. Patients with PDAC are randomized to arm A (conventional procedure) or arm B (isolation procedure) during DPS. The ISOP-DP trial is conducted in 11 Japanese high-volume centers (Additional file 1, institution list) that have been board-certified as training institutions by the Japanese Society of Hepato-Biliary-Pancreatic Surgery. To ensure the high quality of the study, interventions are performed by instructors and expert surgeons certified by the society. This study aims to evaluate the oncological and surgical benefits of the isolation procedure during DPS for patients with PDAC in comparison with the conventional procedure. This study is therefore designed to evaluate the superiority of the isolation procedure (arm B) compared with the conventional procedure (arm A) during DPS in terms of a 2-year recurrence-free survival (RFS) rate. All patients are required to undergo postoperative examination every 3 months for at least 2 years after surgery. Signs of suspected disease recurrence will be closely monitored. The schedule of this trial is shown in Fig. 1. The study period of the ISOP-DP trial is expected to be 4 years, including 2 years for patient recruitment and 2 years of follow-up.

### Study endpoints

The primary endpoint is the 2-year RFS rate. Secondary endpoints include operative time, intraoperative blood loss volume, and transfusion; incidence of postoperative complications within 90 days after surgery (including grade B or C pancreatic fistula, delayed gastric emptying (DGE), and intra-abdominal hemorrhage); all-morbidity rate within 90 days after surgery; and mortality rate within 90 days after surgery. We also include the rates of R0 and R1, number of harvested lymph nodes, number of metastatic lymph nodes, lymph node ratio, overall survival (OS) time, RFS time, and local recurrence rate. R0 status is defined as the absence of tumor cell infiltration within 1 mm of the resection margin, and R1 status is defined as the presence of tumor cell infiltration within 1 mm of the resection margin [13]. Postoperative pancreatic fistula (POPF) [14], DGE [15], and intra-abdominal hemorrhage [16] are defined and graded according to the International Study Group of Pancreatic Surgery (ISGPS) criteria. Postoperative complications other than POPF, DGE, and intra-abdominal hemorrhage are graded by the Clavien-Dindo classification [17]. Postoperative diarrhea is graded according to the Common Terminology Criteria for Adverse Events version 4.

### Statistical analysis

#### Sample size

Clinical trials of isolation and conventional procedures during distal pancreatectomy for pancreatic body/tail cancer have not been widely reported. A two parallel multicenter randomized selection phase II trial compared the conventional procedure with isolation procedure as a basis for a future phase III trial [18]. Its principal aim was to evaluate whether the isolation procedure has a better 2-year recurrence-free survival rate than the conventional procedure in DPS for PDAC. In an observational study, Abe et al. reported that the recurrence rate during the approximately 2-year follow-up period of the conventional procedure was 75%, whereas that of the isolation procedure was 67% [7]. In the current study, we also assume that each group will have a similar 2-year progression-free survival rate. We therefore calculated the number of patients required on the basis of a selection design so that the conventional procedure arm would select a regimen with a 2-year recurrence-free survival rate 8% higher than the expected value with 80% probability. The minimum required number of cases is 46 cases per group. Assuming that approximately 10% of ineligible cases will occur, the target number of registered cases is set to 50 per group (100 cases in total).

#### Statistical analysis plan

The primary population for efficacy analysis will be the intention-to-treat population, defined as all randomized patients. The primary endpoint is the 2-year RFS rate. The primary analysis will be based on the full analysis set (FAS), which consists of all randomized patients except those found to be ineligible after enrollment. The Kaplan-Meier method will be used to estimate the progression-free survival curve and 2-year progression-free survival rate. The regimen with a higher 2-year progression-free survival rate will be considered as the more promising procedure of the two. The Brookmeyer-Crowley method will be used to calculate the 95% confidence interval for the 2-year progression-free survival rate. Although the design of choice cannot confirm the results based on a hypothetical test, it would compare the progression-free survival curves using the log-rank test as a reference. A Cox proportional hazard model will be applied to calculate HRs and 95% confidence intervals adjusted for stratification factors (i.e., approach method and preoperative adjuvant therapy). For evaluation of secondary endpoints, categorical outcomes will be summarized using frequency and percentage for each arm and will be compared using Fisher’s exact test. Mann-Whitney-Wilcoxon test will be used to compare continuous outcomes and will be summarized using the median and interquartile range.

#### Study population

Table 1 shows a detailed overview of all eligibility criteria. Patients are eligible if they meet the ISOP-DP trial definitions for resectable PDAC and no contact with the portal vein (PV) and superior mesenteric vein (SMV), and are scheduled to undergo DPS. Resectable PDAC is defined according to the *Classification of Pancreatic Carcinoma of Japan Pancreas Society (Fourth English Edition)* [19] and the National Comprehensive Cancer Network (NCCN) definition 2020 [20]. Patients eligible for inclusion in this study are those undergoing open or laparoscopic DPS for pancreatic body and tail cancer after excluding those with intra-ductal papillary mucinous neoplasm, neuroendocrine tumors, mucinous cystic neoplasm, or metastatic pancreatic tumors or similar. In addition, simultaneous resection of the pancreatic parenchyma and splenic vein in one session will be rendered possible through evaluation of preoperative imaging study findings. Patients indicated for spleen-preserving distal pancreatectomy (SPDP) and those indicated for the Warshaw operation (spleen-preserving and splenic artery/vein resection) will be excluded.

**Table 1:** Eligibility criteria

Inclusion criteria	Exclusion criteria
(1) Patients who have been diagnosed with resectable pancreatic cancer (adenocarcinoma, adenosquamous cell carcinoma, mucinous carcinoma, and anaplastic carcinoma) according to the *Classification of Pancreatic Carcinoma of Japan Pancreas Society (Fourth English Edition)* [18] and the *National Comprehensive Cancer Network (NCCN) definition 2020* [19], excluding invasive intraductal papillary mucinous carcinoma (IPMC) by image diagnosis at the initial diagnosis, and for whom body-tail pancreatectomy or tail pancreatectomy is planned. Preoperative biopsy is not mandatory, however, and allows clinical diagnosis. (2) ASA-PS (American Society of Anesthesiology, General condition classification) is Class 1–3. (3) Age over 20 years. (4) Patient has sufficient judgment to understand the content of the research and has provided written consent.	(1) Patients who have not been diagnosed with resectable pancreatic cancer by image diagnosis as the initial diagnosis. (2) Patients with tumor suspected of portal vein (superior mesenteric vein) invasion. (3) Patients with severe ischemic heart disease. (4) Patients with cirrhosis or active hepatitis requiring treatment. (5) Patients with dyspnea requiring oxygen administration. (6) Patients undergoing dialysis due to chronic renal failure. (7) Patients with tumor for which arterial reconstruction of the superior mesenteric artery, common hepatic artery, celiac artery, etc. is considered necessary. (8) Patients with strong suspicion of paraaortic lymph node metastasis. (9) Patients with active double cancer thought to affect adverse events and prognosis. (10) Patients with usage of long-term oral steroids that may affect adverse events. (11) Patients considered to have potential difficulty participating in the study due to psychosis or psychiatric symptoms. (12) Cases other than invasive pancreatic ductal carcinoma by preoperative biopsy. Invasive pancreatic ductal carcinoma is classified into four types: adenocarcinoma, adenosquamous cell carcinoma, mucinous carcinoma, and anaplastic carcinoma, and invasive intraductal papillary mucinous carcinoma (IPMC) is excluded. However, a preoperative biopsy is not mandatory. (13) Patients who cannot use either iodine and gadolinium contrast agent due to severe drug allergy. (14) Patients whose abdomen makes it difficult to perform the prescribed procedure due to a history of upper abdominal surgery, such as on the stomach, spleen, kidney, liver, transverse colon, or retroperitoneum, including the pancreas and for pancreatitis. (15) Patients who may require resection of organs other than the spleen, left adrenal gland, and gallbladder.

*Abbreviations*: *ASA-PS* American Society of Anesthesiologists-Physical Status

### Randomization

**Fig. 2:**
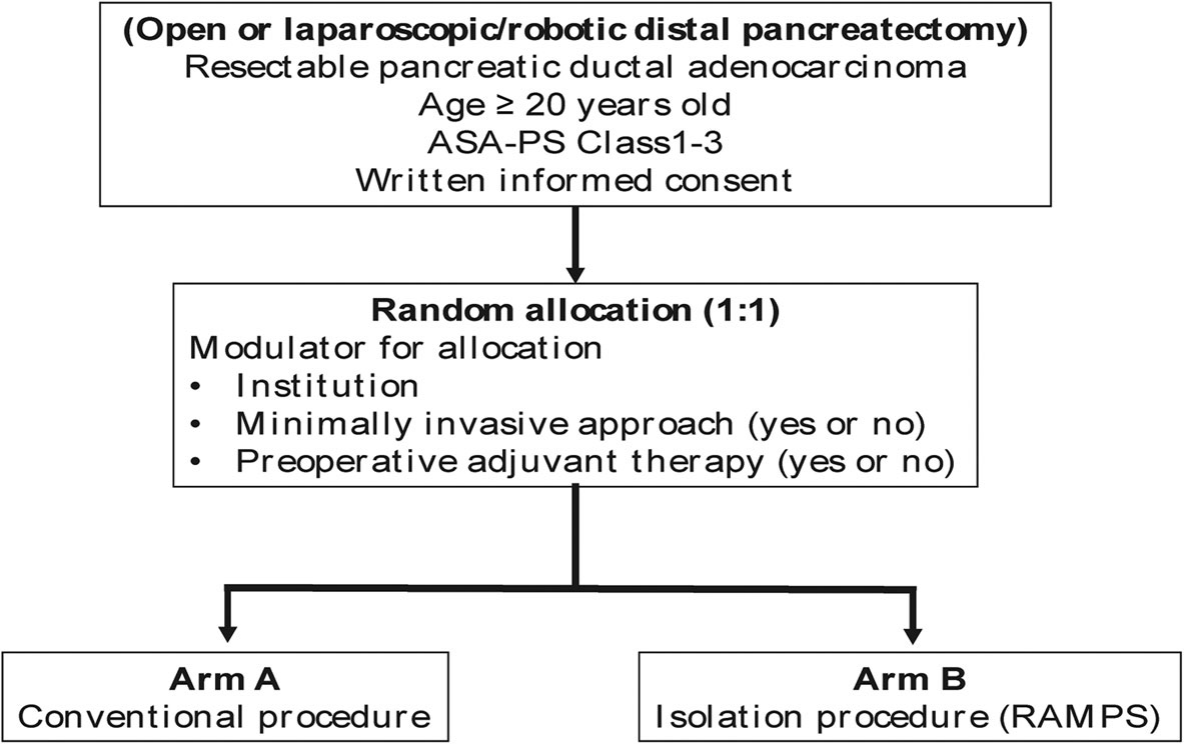
Flow diagram of the ISOP-DP (isolation procedure vs. conventional procedure during distal pancreatectomy) trial

After confirmation of eligibility including written informed consent, patients will be randomized in a 1:1 allocation ratio to either arm A (conventional approach) or arm B (isolation approach) with a random block size. Central randomization and registration will be applied, using UMIN Internet Data and Information system for Clinical and Epidemiological research, cloud version (INDICE cloud). After assessment for eligibility at registration, patients will be centrally randomized to either arm A or arm B. To minimize background bias between the two groups, this study is stratified by participating institution according to use of minimally invasive approach (yes or no), and preoperative adjuvant therapy (yes or no). The indication and regimen for preoperative adjuvant therapies depend on the treatment strategies of the participating institutions. We use Pocock and Simon’s minimization method for random assignment and the Mersenne Twister for random number generation (Fig. 2, flow diagram of the ISOP-DP trial). All patients are blinded to the surgical approach they will receive, and they are required to sign an informed consent form before enrollment in the study. Blinding of the surgeons is not possible because of the different techniques used during the operation. Assessment of the result will be made by one of two independent researchers (A.S., N.I.) who will be blinded to the surgical procedures. Once distal pancreatectomy is performed, there is no difference in postoperative radiographic findings between the two surgical approaches, so it should not affect the radiologist’s assessment.

### Interventions

#### Trial intervention (isolation approach)

In the isolation procedure group, transection of the root of the splenic artery (SA) and the pancreatic transection are performed first, followed by division of the splenic vein (mandatory procedure). At that time, the branch from the splenic artery (dorsal pancreatic artery), the branch to the splenic vein (left gastric vein, inferior mesenteric vein), and the short gastric artery and vein are also disconnected as soon as possible (recommended procedure). An operation to lift up the pancreatic neck from the dorsal portal vein or superior mesenteric artery to expose the splenic vein (so-called tunneling) is allowed. After that, lymph node dissection, such as of hepatoduodenal mesentery (lymph node No.12) [19], and around the common hepatic artery perimeter (lymph node No.8) [19], and lymph node dissection around the SMA (lymph node No.14p) [19] are performed (recommended procedure). At the end of the resection operation, the pancreatic body/tail and the spleen are mobilized and removed (required procedure) by detachment of the retroperitoneum. Strasberg et al. described the division of the left gastric artery as an optional step that may be omitted; otherwise, this procedure is identical to the RAMPS procedure [5].

#### Control intervention (conventional approach)

In the conventional procedure group, first, the pancreatic body and tail and spleen are mobilized by detachment of the retroperitoneum (mandatory procedure). The regional lymph nodes of the pancreatic body/tail, such as the hepatoduodenal mesentery (lymph node No. 12) [19] and the common hepatic artery perimeter (lymph node No. 8) [19], are removed (recommended procedure) and dissection of lymph nodes (lymph node No.14p) [19] around the SMA (recommended procedure). After dissection of the gastro-splenic ligament and pancreatic transection, the splenic vein is divided at the end of the resection procedure (required procedure), although pancreatotomy or division of the SA in early phases is allowed to prevent bleeding and secure a safe field of view.

#### Common to both groups (allowable procedure)

1. In both groups, the spleen is resected with the pancreas. The direction of the detachment of the pancreatic body/tail from the retroperitoneum is recommended to proceed from the right side to the left side. Lymph node dissection shall be regional lymph node dissection (lymph node Nos. 8a, 8p, 12a, 12p, 11p, 11d, and 14p) in the *Classification of Pancreatic Carcinoma of Japan Pancreas Society (Fourth English Edition)* [19] (recommended procedure). As a general rule, the nerve plexus around the SMA is preserved all around (recommended procedure).
2. In both groups, the approach of open surgery, laparoscopic surgery, and robotic surgery is left open to the decision of the operators.
3. To ensure safety, conversion from laparoscopic or robotic surgery to open surgery based on the intraoperative findings such as uncontrollable bleeding or severe adhesion is acceptable. However, the conversion from open surgery to laparoscopic or robotic surgery is not specified.
4. For safety, with consideration of bleeding prevention, it is permissible to perform adhesion detachment around the spleen.
5. Hemostasis (compression, suturing, branch transection) is permissible if there is bleeding from portal veins.
6. The method of pancreatic transection or the method of closing the pancreatic stump is left open to the decision of the operators.
7. Splenic vein resection together with the pancreatic parenchyma after isolation of the parenchyma during DPS is acceptable if it can be safely performed.
8. Combined resection of the surrounding organs other than the spleen (stomach, large intestine, etc.) is allowable. However, if the surgical procedure is changed to portal vein resection, celiac artery resection, exploratory laparotomy, etc. during the operation, it will be considered as a deviation, and the protocol treatment will be stopped according to the criteria for discontinuing protocol treatment. Patients with protocol deviation must be followed in an intention-to-treat analysis.
9. If essential procedures cannot be performed in either group, it is considered to be a deviation and the protocol treatment is stopped according to the criteria for discontinuing protocol treatment. Whether or not the recommended procedure is performed is not treated as a target of deviation judgment.

### Standardization and validation of interventions

To guarantee the quality of both procedures, board-certified surgeons from the Japanese Society of Hepato-Biliary-Pancreatic Surgery perform or supervise all surgery. Both procedures are performed in daily practice depending on the case. Before the start of the ISOP-DP trial, all participating surgeons in this trial held consensus meetings to determine the details of the operative techniques for both groups by observing and discussing several operative videos to ensure standardization of the interventions in all institutions at which more than 50 pancreatectomies for pancreatic cancer were performed per year, in accordance with the Japanese Pancreatic Cancer Treatment Guidelines. On the basis of the meetings, we determined that a photographic record after pancreatic transection is necessary for both groups for validation of intervention quality, because the operative techniques of this approach have been certified by the Japanese Society of Hepato-Biliary-Pancreatic Surgery. Central judgment will be conducted for both groups, and the intraoperative images are evaluated to determine whether the procedure is performed according to the protocol by three reviewers (two independent surgeons and principal investigator); cases with discrepancies are re-evaluated simultaneously by the three reviewers until a consensus is reached. If there is disagreement among the three reviewers, the majority classification is chosen to settle whether the procedure has been performed according to the protocol. If it is determined that the procedure is not performed according to the protocol, it will be treated as a case that deviates from the protocol.

### Recruitment

To achieve adequate participant enrollment to reach the target sample size within the study period, 11 Japanese high-volume centers will participate in the ISOP-DP trial.

### Follow-up

After randomization, the patients will be followed every 3 months, or more often if the patient’s situation requires it, for at least 2 years. Patients in this study will undergo plain/enhanced computed tomography every 3 months to assess postoperative recurrence and metastases. Patients with iodine allergies will undergo plain computed tomography plus enhanced magnetic resonance imaging. The accuracy of the latter study method is not inferior to that of the former [21, 22]. RFS is defined as the time from operation to the time of finding any recurrence or metastasis or until death. OS is defined as the time from operation to the time of the last follow-up or death.

### Data and safety monitoring

During the study, an independent data monitoring committee (Clinical Study Support Center, Wakayama Medical University) will monitor the safety of the trial subjects by qualitative analyses of feasibility, accrual rate, and adverse events, as well as dropouts every 6 months. Data are collected via a case report form using paper and stored and managed securely by the data monitoring committee. To improve the quality of data, after written consent is signed, all baseline assessments will be conducted before randomization. The handling of all cases is managed by subject identification code or anonymized registration number. The correspondence table of the anonymizing codes and names, as well as the consent form containing the name, is kept in separate restricted-access lockable document storage at each participating institution. To promote data quality, missing data will be pursued until received or confirmed as unavailable, or until the trial reaches analysis. No interim analyses are planned in the ISOP-DP trial. The principal investigator has the right to terminate the trial at any time in consultation with the biostatistician. The trial would be terminated if the incidence or severity of adverse events in the trial indicated a potential health hazard caused by the study treatment. The trial is terminated if patient enrollment appears unsatisfactory with respect to quality or quantity, if data recording is severely inaccurate or incomplete, or if external evidence renders it necessary. The procedure for deciding to discontinue the entire study is as follows: the principal investigator must request a review by the Wakayama Medical University Ethics Review Board and make a report. If the principal investigator decides to discontinue the entire research, the principal investigators of the other institutions will be immediately notified of the reason and subsequent actions. The principal investigator of the other institutions contacted informs the subjects of the discontinuation of the entire research and the reason and takes appropriate action immediately. All adverse events observed by the investigators will be recorded up to 90 days after surgery and reported to the principal investigator and clinical trial coordination center. The assignment of the severity or grading should be made by the investigator responsible for the care of the participant. Serious adverse events are defined as those that are life-threatening or result in death. Serious adverse events will be collected and recorded according to good clinical practice throughout the study period. The patients enrolled in this study will receive standard-of-care supportive measures and all other medically necessary interventions as needed.

### Ethics

#### Research ethics approval

This study is performed in accordance with the Declaration of Helsinki. The protocol has been approved by the Wakayama Medical University Hospital Ethics Committee (approval number 2986). The trial protocol has also been registered in the protocol registration system at ClinicalTrials.gov (NCT) and the University Hospital Medical Information Network Clinical Trials Registry (UMIN). All patients will be scheduled only after comprehensive information concerning the nature, scope, and possible consequences of the clinical trial have been provided to them in an understandable way by the investigator. Written informed consent to inclusion in the study will be obtained from each patient before the operation. The procedure, benefits, risks, and data management of this study will be clarified in detail for the patients during the preoperative conversation.

#### Dissemination policy

The results of the ISOP-DP trial will be submitted to a peer-reviewed journal and will be presented at national and international conferences, regardless of the trial outcomes. Authorship will be agreed in accordance with the ISOP-DP trial publication policy and in line with international guidelines.

## Discussion

The oncological impact of the isolation by the so-called non-touch technique remains controversial in digestive surgery [1–4, 23]. The outcome could be affected by anatomical features of vessels connected to organs in which cancer has developed, or by minimal invasiveness of manipulation with laparoscopic/robotic forceps. Although Strasberg et al. may have intended to report the RAMPS procedure with focus on the number of lymph nodes dissected and the higher R0 resection rate, this procedure also has potential as an isolation procedure for the pancreato-splenic area by division of the splenic vessels and transection of the pancreas in the early stages of surgery. The RAMPS procedure has been reported to demonstrate a significantly higher R0 resection rate and a higher number of lymph node dissections than standard DP [5, 6]. Meanwhile, in a recent systematic review and meta-analysis, there were no significant differences in recurrence rate, OS, or DFS [11]. In other systematic reviews and meta-analysis, the RAMPS procedure was shown to have better surgical outcomes (time and bleeding volume) and pathological outcomes with a significantly higher R0 resection rate and a larger number of lymph node dissections than the standard DP [12]. Otherwise, there were no significant differences in postoperative pancreatic fistula, postoperative complications, length of stay, or mortality. There was no significant difference in the postoperative recurrence rate, but the only finding of favorable survival outcome was 1-year postoperative OS with the RAMPS procedure. Surprisingly, in these systematic reviews and meta-analysis, all studies analyzed were retrospective, and many of them were historically controlled studies in which surgery was performed by the RAMPS method in recent years when surgical techniques and devices were developed. The most important endpoint in the treatment of pancreatic cancer is OS, although what exactly contributes to the extension of OS in RAMPS procedures is unclear and remains controversial.

In an international multicenter study, Korrel et al. reported that Gerota’s fascia resection, R0 resection, and reduction in LNR were factors associated with improved overall survival in DP-treated PDAC [24, 25], but the surgical approach was not included as a risk factor in their analysis. There is a possibility that the squeezing out of tumor cells can be reduced by a surgical approach. It has become possible in recent years to observe and manipulate the deep abdominal cavity and the retroperitoneal space in detail with a laparoscopic field of view and developed surgical devices. As a result, the oncological impact of the isolation technique may be greater than the difference in the direction of the retroperitoneal dissection. If there is an oncological benefit in the RAMPS procedure, it may be due to early blockage of the lymphatic, arterial, and portal venous system in the pancreatic body/tail by prioritizing the vascular dissection in the excision phase. However, the oncological benefits of this surgical procedure have not yet been demonstrated in prospective randomized controlled trials.

If the ISOP-DP trial shows that the isolation procedure can improve recurrence-free survival with a similar R0 rate and a similar number of lymph node dissections to the conventional procedure, the isolation procedure is expected to become a worldwide standard procedure during DPS for PDAC under the concept of isolation technique. Otherwise, if there are no significant differences in endpoints between the groups, it will demonstrate that both procedures may be justified from surgical and oncological points of view.

## Trial status

The ISOP-DP trial was opened in October 2020. At the time of submission of this paper (January 2021), the protocol is version 1.0. The completion date is estimated to be September 2025.

## Supplementary Information

**Additional file 1.** Institution list

## Data Availability

The datasets used and/or analyzed will be available from the corresponding author on reasonable request after the current study is complete.

## Abbreviations

RAMPS: Radical antegrade modular pancreatosplenectomy
PDAC: Pancreatic ductal adenocarcinoma
INDICE cloud: UMIN Internet Data and Information system for Clinical and Epidemiological research, cloud version
NCCN: National Comprehensive Cancer Network
OS: Overall survival
DPS: Distal pancreatosplenectomy
POPF: Postoperative pancreatic fistula
DGE: Delayed gastric emptying
PV: Portal vein
SMA: Superior mesenteric artery
SMV: Superior mesenteric vein
