# Supplementary material for: ISOlation Procedure vs. conventional procedure during Distal Pancreatectomy (ISOP-DP trial): study protocol for a randomized controlled trial
Source: Trials. 2021 Sep 16;22:633. doi: 10.1186/s13063-021-05523-y (PMC8447574; doi:10.1186/s13063-021-05523-y)
Supplement: Supplementary file 1 — Additional file 1. Institution list [file 13063_2021_5523_MOESM1_ESM.docx]

Additional file 1, institution list

| **Name of institution** |
| --- |
| Wakayama Medical University, School of Medicine. |
| Faculty of Medicine, Academic Assembly, University of Toyama. |
| Nara Medical University. |
| Shiga University of Medical Science. |
| Kindai University Hospital. |
| Nagoya Central Hospital. |
| Osaka City University, Graduate School. |
| Kobe University Graduate School of Medicine. |
| Graduate School of Medical Sciences, Kumamoto University. |
| Jikei University School of Medicine. |
| Nagoya University, Graduate School of Medicine. |
